# The effect of self-selected versus standardised warm-ups on kayak ergometer performance in Hungarian elite kayakers: a randomized controlled crossover trial

**DOI:** 10.1186/s13102-026-01555-6

**Published:** 2026-02-05

**Authors:** Emese Trájer, Péter Katona, Brigitta Kovács, Ádám Suskó, Márton Rakovics, Tímea Kováts

**Affiliations:** 1 Hungarian University of Sports Science, Budapest, Hungary; 2 Katalin Kovács National Canoe Sports Academy, Sukoró, Hungary; 3 Centre for Translational Medicine, Semmelweis University, Budapest, Hungary; 4 Faculty of Social Sciences, Department of Statistics, ELTE Eötvös Loránd University, Budapest, Hungary; 5 Department of Sports Medicine, Semmelweis University, Budapest, Hungary

**Keywords:** Warm-up strategies, Sprint kayaking, Exercise performance, Self-selected warm-up

## Abstract

**Background:**

Warm-up strategies play a crucial role in enhancing athletic performance and preventing injuries, yet the optimal warm-up protocol for sprint kayaking remains unclear.

**Methods:**

This study compared the effects of four different warm-up approaches—interval, increasing intensity, continuous, and self-selected—on performance in a 2-minute maximal kayak ergometer test among eleven elite male sprint kayakers. Each participant completed all warm-up types in a randomized order, followed by a 2-minute all-out time trial. Performance power output, heart rate, blood lactate levels, and subjective ratings of the warm-ups were measured.

**Results:**

Results showed that the interval warm-up produced the highest average power output, significantly outperforming the increasing intensity warm-up. However, performance after the self-selected warm-up was not significantly different from the interval warm-up, and athletes rated the self-selected warm-up significantly better than the other warm-ups. Higher blood lactate concentrations measured immediately before the time trial were strongly associated with reduced performance regardless of warm-up type. The increasing intensity warm-up resulted in the highest pre-trial lactate levels and lowest performance.

**Conclusions:**

These findings suggest that while structured interval warm-ups can enhance performance, self-selected warm-ups are appreciated by experienced sprint kayakers and can be implemented if blood lactate level is not excessively elevated. This study highlights the importance of balancing warm-up intensity to maximize performance without inducing premature fatigue.

**Trial registration:**

Not applicable. This study did not involve a health care intervention and did not meet the criteria for clinical trial registration.

**Supplementary Information:**

The online version contains supplementary material available at 10.1186/s13102-026-01555-6.

## Introduction

Warm-up strategies are critical components of athletic training, aiming to enhance performance [1–4] and prevent injuries [5, 6]. However, the underlying mechanisms and optimal design of warm-ups remain debated, especially in sports like kayaking, where performance depends on strength, endurance and technique. In this context, identifying evidence-based, sport-specific warm-up methods is crucial for maximizing competitive readiness.

The possible performance-enhancing effect of pre-competition warm-ups has been attributed to different physiological changes, including increased muscle and core temperature, induced metabolic and cardiovascular changes, enhanced neuromuscular activation, and provoked psychological effects [1, 3, 4, 7]. Apart of these beneficial influences, active warm-up, depending on its intensity, could deplete energy sources and cause fatigue as well [8]. However, phosphocreatine store can be promptly filled up, as well as imbalances in H^+^ ion concentration can be quickly reinstated during rest. Thus, not only the volume, intensity, and technique of the active warm-up, but the length of the recovery between the warm-up and the event define the actual performing enhancement effect. Since the advantageous effects of the warm-up might remain longer than the recovery of the deleterious effects might take. Recent scientific literature has identified this phenomenon as post-activation performance enhancement (PAPE) [4, 9].

While research on warm-up strategies in kayak-canoeing remains limited [10–12], insights from comparable sports that rely on both endurance and strength—such as swimming and cycling—suggest that effective warm-ups typically include a low-intensity, aerobic component followed by brief high-intensity efforts [3]. However, the characteristics of the high intensity part remain unclear and likely require sport-specific investigation.

In water-based sports like swimming, triathlon, and sprint kayaking, warm-up routines often combine both land- and water-based components [13–16]. Among sprint kayakers, dryland warm-ups typically include passive and dynamic stretching, self-massage, stationary cycling, as well as strength and activation exercises, generally lasting 10–20 min [16].

Although several studies have examined on-water warm-up strategies in sprint kayaking [1, 5, 10, 12, 16], there are no clear recommendations for the current on-water warm-up protocol in competitions. Over the past two decades, laboratory-based research has explored the effects of different warm-up intensities on kayak ergometer performance. Bishop et al. [10] found that overly intense continuous warm-ups could impair subsequent performance due to increased metabolic acidaemia. In a follow-up study, they reported that intermittent high-intensity warm-ups enhanced oxygen uptake without reducing anaerobic capacity, thereby improving supramaximal performance [11]. However, Dingley et al. [12] compared self-selected and standardized warm-ups, noting that performance metrics remained consistent regardless of the warm-up type. These conflicting findings over the past two decades show that it is still uncertain whether a prescribed warm-up protocol is better than one chosen by the athlete. While Bishop et al. [11] found that interval warm-up is the most effective before sprint kayaking events, Dingley et al. [12] concluded that it can be left to the athletes to decide which warm-up to perform before the competition as there was no difference in effectiveness between tailored interval and self-selected warm-ups. Considering the result of the latter, it might even be a rather appealing approach for highly experienced, international-level kayakers that the athlete „feels” which warm-up strategy is the most effective for him/her individually. However, to accept or refuse this hypothesis, it needs additional objective results. Thus, our goal was to compare self-selected warm-ups with specifically tailored protocols (interval, continuous, increasing-type) and to determine whether we can rely on the athlete’s subjective feeling about the effectiveness of the warm-up protocol. Accordingly, our study hypothesized that self-selected warm-ups would not be inferior to standardized warm-ups in terms of sprint kayak performance outcomes.

## Methods

### Participants

12 adult elite Hungarian male sprint kayak athletes competing on elite and international level [17] were selected on the basis of their level of K1-500 m time and willingness to participate in the study. Due to an upper respiratory infection, the final number of participants was 11 (age 20.5 ± 1,72 years, body mass 83.30 ± 9.8 kg, height 182.43 ± 7.93 cm). All of them were all members of the Under-23 (U23) national team. Anthropometric and body composition measurements are summarized in Table 1.

**Table 1:** Descriptive statistics of the participants (*N* = 11)

Variable	Mean (± SD)	Minimum	Maximum
Age (y)	20.5 (± 1.7)	18.0	24.0
Weight (kg)	83.3 (± 9.8)	69.2	96.2
Height (cm)	182.4 (± 7.9)	170.8	194.0
BMI	25.0 (± 1.9)	22.0	29.1
Muscle mass (kg)	40.6 (± 4.7)	33.1	46.5
Body fat %	13.8 (± 3.6)	10.1	22.9
Muscle %	48.8 (± 2.2)	43.2	51.2

They were all familiar with exercising on kayak ergometer and were currently in off-season in winter. All participants were encouraged to undertake their normal training and diet, except that they were asked not to eat 2 h before and not to train on the day before the tests.

After all participants were informed about the study design and the risks, they completed a written informed consent. Ethical approval was obtained from the Research Ethics Committee of the Hungarian University of Sports Science (MTSE-OKE-KEB/08/2023).

### Experimental design

At the beginning of the study, all participants underwent an anthropometric assessment, including measurements of body weight (kg), height (cm) and body composition: percentage of muscle and body fat, and muscle mass was measured using a multifrequency bioelectrical impedance analyser (Tanita BC-418MA, Tanita Europe B.V., Amsterdam, The Netherlands). Afterwards, all participants performed a graded exercise step-test (GXT) on kayak ergometer (Old Danube, Szentendre, Hungary) in order to determine their maximal performance (P_max_), maximal heart rate (HR_max_), and the individual physiological thresholds.

Tests were conducted in a randomized, crossover, counterbalanced order [18] The eleven participants were divided into four groups. All 11 athletes performed the four different warm-ups followed by a 2-min time trial (TT) over four consecutive weeks, on the same day and at the same time, but in different sequences according to their group assignment. Thus, for each participant, tests were separated by 7 days. During the tests, lactate values after the warm-up and after the TT, average power output during the TT, peak heart rate during the TT, and athletes’ subjective ratings of the different warm-up types (on a 1–5 scale, with 5 being the best) were recorded.

GXT and testing sessions were all conducted in controlled laboratory settings, at room temperature, with controlled air humidity (50–70%), during the morning hours (9:00 a.m.–12:00 p.m.).

### Kayak ergometer

All physiological testing was performed on a calibrated, wind-braked kayak ergometer (Old Danube, Szentendre, Hungary). The footrest and the seat of the ergometer were adjusted by the athletes before each test. The ergometer was interfaced with a software (ArguStress. Inc., Budapest, Hungary) that measured, calculated, and stored the current and the accumulated work, speed, heart rate and stroke rate. Participants could also follow their current momentary power output and HR on a monitor real-time, which allowed them to maintain the required level of intensity.

### Heart rate

Heart rate was monitored using a Polar sensor that collected data beat by beat (Polar H10, Kempele, Finland) and was interfaced with the data analysis software (ArguStress. Inc., Budapest, Hungary). During the GXT, the average heart rate of the last 30 s of each stage was calculated. Peak heart rate values were obtained from the beat-by-beat analysis by identifying the highest measured value, with particular attention given to potential outliers.

### Blood analysis

Arterialized capillary blood (20 µL) was sampled from hyperaemic earlobe. Nicoflex (Medimpex Kereskedelmi Zrt., Budapest) was used on the earlobe 5 min before the first warm-up as a cutaneous vasodilator. Capillary blood samples were taken before and after the dry-land warm-up, before the ergometer warm-up (WU) period at rest, 1 min before the start of the 2-min TT test, and 1,2,4,6 min after the TT test. Blood samples were taken every other minute until the values started to reduce. Capillary blood samples were measured with a lactate analyser (Biosen C-line, EKF, Cardiff, UK).

### Graded exercise test

2 weeks before the warm-up testing, each participant underwent a GXT on kayak ergometer until voluntary exhaustion. The GXT was used to determine the P_max_, HR_max_ and the power and HR at their physiological thresholds (lactate threshold, LT; anaerobic threshold, AT; individual anaerobic threshold, IAT). After a guided 10-minute dry land warm-up each of them started with a workload of 60 watts (W) and increments of 30 W were applied at 2-minute intervals. 1-minute resting time between the steps allowed for sampling capillary blood from the earlobe to measure lactate. The test continued until the participants could no longer maintain the required power output. HR was continuously monitored by Polar H10. After the test, Ergonizer software (^®^1991–2017, K. Roecker) was used to determine their individual thresholds. LT and AT were calculated using the Dickuth method [19], and IAT was calculated using the modified D_max_ method [20].

### Warm-up procedures

#### Dry-land warm up

The approximately half-hour (25-minute) - dry-land and ergometer - warm-up was determined based on a preliminary assessment. All athletes performed all the four different types of warm-ups WU followed by a 2-min TT. Before each WU style, but the self-selected one, participants had to perform a guided, 10-min dry-land warm-up up which was developed specifically for kayakers (Appendix A).

All guided WUs on ergometer took 13 minutes and consisted of three parts: a 2-min „easy” paddling, without any special instruction, while the drag of the ergometer had to be on the first 2 easiest levels. It was followed by a 5-min paddling when HR had to be maintained between the HR at LT and IAT. The last 6-min paddling differed as follows:

#### Continuous style warm up (Continuous WU)

The aim was that neither HR nor intensity (watts) increase above the values of IAT. Participants had to maintain a target HR, which was their HR at AT. If HR approached their HR at IAT or HR at LT, they were instructed to decrease or increase the watt, respectively.

#### Interval style warm up (Interval WU)

The aim was to increase the intensity (watts) but not the HR. In order to reach that goal, athletes had to perform 6 intervals. The work/rest ratio was 8–10 s / 50 s. The intensity of the intervals was calculated from the last step of the GXT, thus, kayakers had to reach the 85-90-95-100-80% of their maximal intensity (the intensity of the last step of the GXT) during the 8–10 s interval. Athletes were instructed to reach the required intensity as fast as it was possible, to mimic a race start. However, HR should remain below the HR of the IAT throughout the course of the interval phase.

#### Increasing style warm up (Increasing WU)

The aim was to increase both the intensity (watts) and the HR. Athletes had to perform 4 increasing repetitions. The targeted intensity was calculated as the 90-95-100-90% of their maximal intensity of the GXT, respectively. The targeted watts had to be reached in 10 s and hold for 30 s. In between, a 50 s resting period with a very low intensity paddling was inserted. The aim of this WU was to reach at least once the 95% of HR_max_ while the HR should decrease below the AT during the 50 s rest.

#### Self-selected warm up (Self-selected WU)

Participants were encouraged to do their own WU that they would typically complete prior to competition. The athletes were given a start time, just as in competition, and were allowed a maximum of half an hour for dry-land and ergometer warm-up, with the ratio between the two left to their discretion. Although the specific description of the individual warm-ups is beyond the scope of the present study, athletes typically performed their dry-land warm-ups in the gym mainly completing upper-body bodyweight exercises (pullups, push-ups, etc.), while sport-specific WU on the ergometer typically consisted of a low intensity continuous paddling with a few intermittent higher intensity insets.

#### Two-minute time trial (TT)

Each WU was followed by a 2 × 1-min rest on the ergometer. Although this is rather on the short end since optimal recovery time is approximated around 5 min in the current literature [9], our goal was to approximate race-like situations, thus, recovery time was chosen based on the consultation with the Hungarian Canoe Federation.

After the first minute of recovery, capillary blood was taken from the earlobe to measure the lactate level at start (La_s_). During the subsequent 1 min, athletes were not allowed to maintain any movement on the ergometer, to simulate the start at a competition. Hereafter athletes completed a 2-min all-out test on kayak ergometer. This length was chosen in order to be comparable with a 500 m kayak TT. Athletes had to reach their best performance during these 2 min. After the test, lactate was measured every 2 min until the result of the next measurement was lower than the previous one to detect peak lactate concentration (La_peak_). HR was also monitored during restitution.

#### Subjective rating of the warm-ups

A 5-point Likert scale was used as a rating of the different warm-up styles [21]. After the 2-min TT, kayakers were asked to give a number between 1 and 5 (5 being the best) as a subjective rating of the different WUs.

### Statistical analysis

Descriptive statistics were used to characterize the study population, and mixed-effects linear models [22] to assess differences in warm-ups accounting for measurements nested within individuals. As the comparison of WU styles was pre-planned as the primary goal of the study, the EMA (2020) guideline accept unadjusted significance values [23]. Warm-up style and other explanatory variables are included as fixed-effects in the models with random intercepts for individuals (i.e. identifiers of participants as a random-effect). QQ-plots, residuals-versus-fitted and scale–location plots were used for model checking of linear models. Cohen’s d effect size analysis and power analysis [24] were based on estimated effects and variances from the mixed-effects models. Data analysis was conducted in R version 4.3.2 (2023-10-31) [25], using the lme4 package [22].

## Results

In four consecutive weeks, all athletes performed all four different types of WUs followed by a 2 min TT in a randomized, counterbalanced order. The lactate values after the WUs (La_s_) and after the TT (La_peak_), the average power output during the TT, the HR_peak_ reached at the TT, as well as the subjective rating of the different WUs on a scale of 1 to 5 (5 being the best) by the athletes are summarized in Table 2.

**Table 2:** Mean (± SD) results for resting and peak lactate, heart rate and maximal power values recorded during the TT tests by warm-up types

WU style	Lactate at start (mmol/L)	Peak lactate (mmol/L)	TT Performance (watts)	TT HR_peak_	Subjective rating of the WU (1–5)
I. Continuous	3.13 (± 1.12)	13.59 (± 1.98)	229.91 (± 37.72)	193.45 (± 8.86)	2.95 (± 1.19)
II. Interval	2.17 (± 0.88)	14.40 (± 2.29)	237.82 (± 45.88)	191.45 (± 9.29)	4.15 (± 0.84)
III. Increasing	4.93 (± 2.04)	13.96 (± 2.38)	221.50 (± 35.06)	194.90 (± 7.13)	3.00 (± 1.25)
IV. Self-selected	3.28 (± 1.76)	14.09 (± 1.53)	224.60 (± 28.58)	189.70 (± 9.92)	4.32 (± 0.64)

**Fig. 1:**
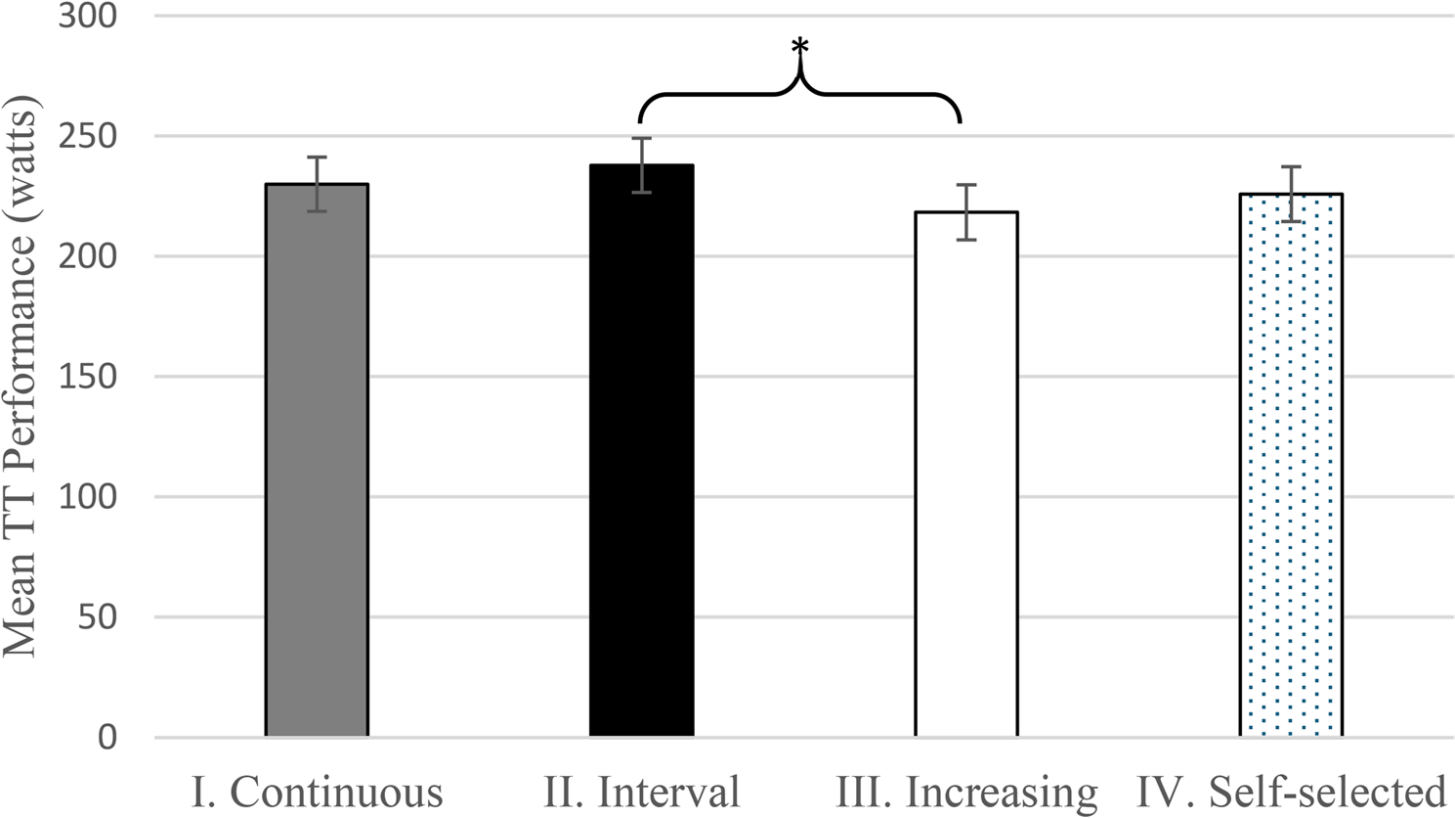
Adjusted mean performance (watts) in the TT test after the Continuous, Interval, Increasing, and Self-selected warm-up types * *p* = 0.019. The complete model output underlying the estimated means and diagnostic plots can be found in Supplementary A

The raw statistics shown in Table 2 should be adjusted for valid comparisons between warm-ups as baselines of athletes are different. After adjustment with random effects models to account for individual baselines, the means of lactate, power, HR, and subjective rating values were found to be significantly different for some of the different WU strategies. The kayakers reached the highest watt values of 237.82 (95% CI 215.75–259.89) after the interval style warm-up, which was significantly higher than the mean performance of 218.32 (95% CI 195.92–240.72) after the increasing style warm-up (Fig. 1). The power of the t-test in the mixed-effects model to detect the observed difference between the Interval and the Increasing WU is 71.6% at the 5% alpha level. To reach 80%, data from 14 athletes would have been needed. See detailed power and sample size estimates in Supplementary E.

**Fig. 2:**
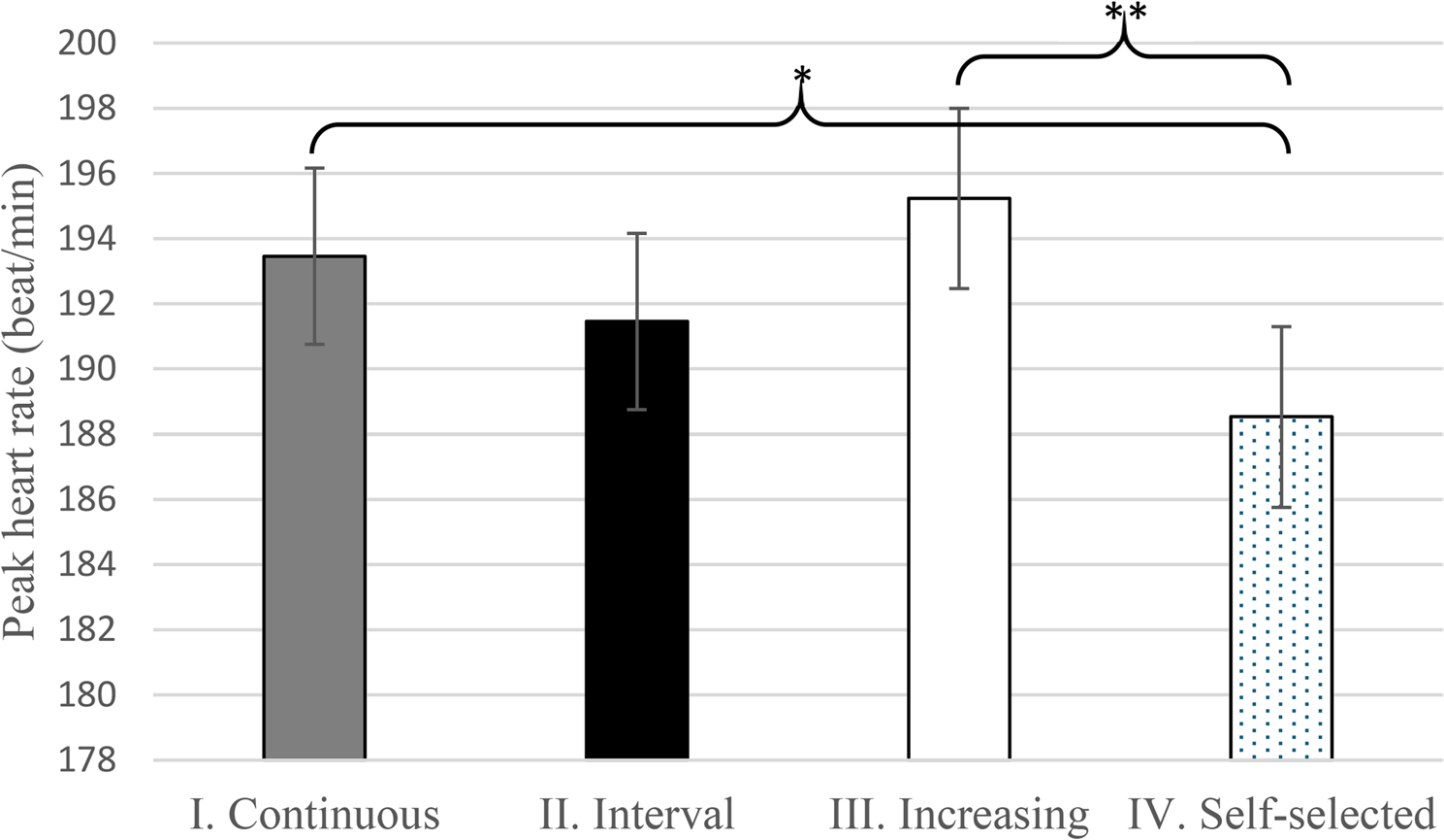
Adjusted peak heart rate during the TT test (mean, +/- SD) the Continuous, Interval, Increasing, and Self-selected warm-up types ** *p* = 0.008, * *p* = 0.040. The complete model output underlying the estimated means and diagnostic plots can be found in Supplementary B

HR_peak_ during the TT test was highest after the Increasing WU with a mean of 195.23 (95% CI 189.81–200.65), and second-highest after the Continuous WU with a mean of 193.46 (95% CI 188.15–198.76). Both were significantly higher than after the Self-selected WU which had a mean of 188.53 (95% CI 183.11–193.95) (Fig. 2).

**Fig. 3:**
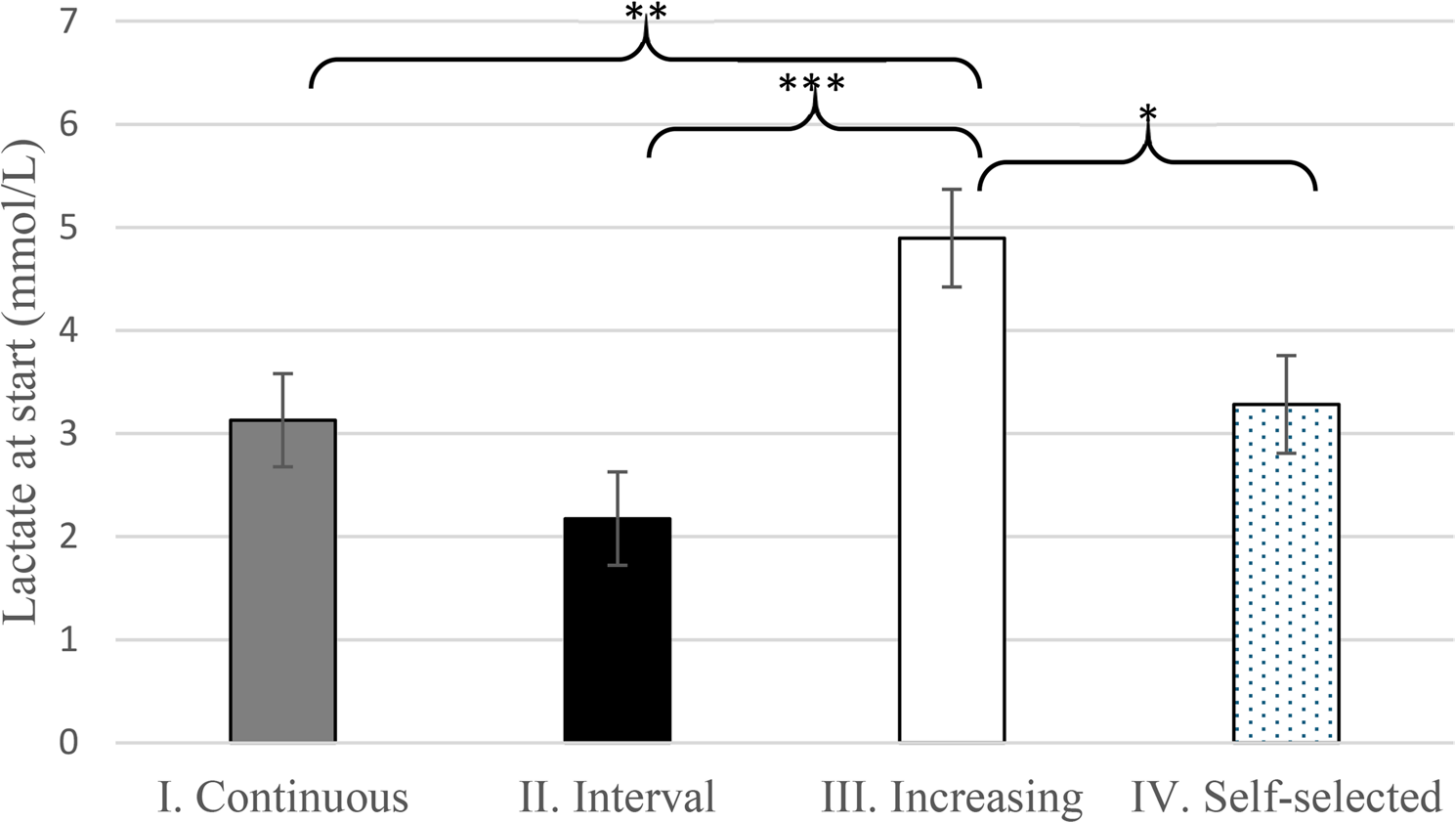
Adjusted lactate at the start (mean, +/- SD) of the TT test after the continuous, interval, increasing, and self-selected warm-up types *** *p* < 0.001, ** *p* = 0.007, * *p* = 0.015. The complete model output underlying the estimated means and diagnostic plots can be found in Supplementary C

Increasing WU resulted in a significantly higher mean starting lactate of 4.90 (95% CI 3.97–5.82) compared to other WU types. The lowest mean starting lactate was 2.17 (95% CI 1.29–3.06) after the Interval WU (Fig. 3). The estimates of the mixed-effects model for lactate at start are shown in Table 3.

**Table 3:** Mixed-effects linear model for lactate at start (mmol/L). Fixed-effects: WU style; random-effects: individual

Variable	Estimate	SE	d.f.	t-value	*p*-value
II. Interval – reference	2.17	0.45	36.12		
I. Continuous	0.95	0.59	28.27	1.605	0.120
III. Increasing	2.72	0.61	28.78	4.457	< 0.001
IV. Self-selected	1.11	0.61	28.78	1.812	0.080

The complete model output and diagnostic plots can be found in the Supplementary C.

**Fig. 4:**
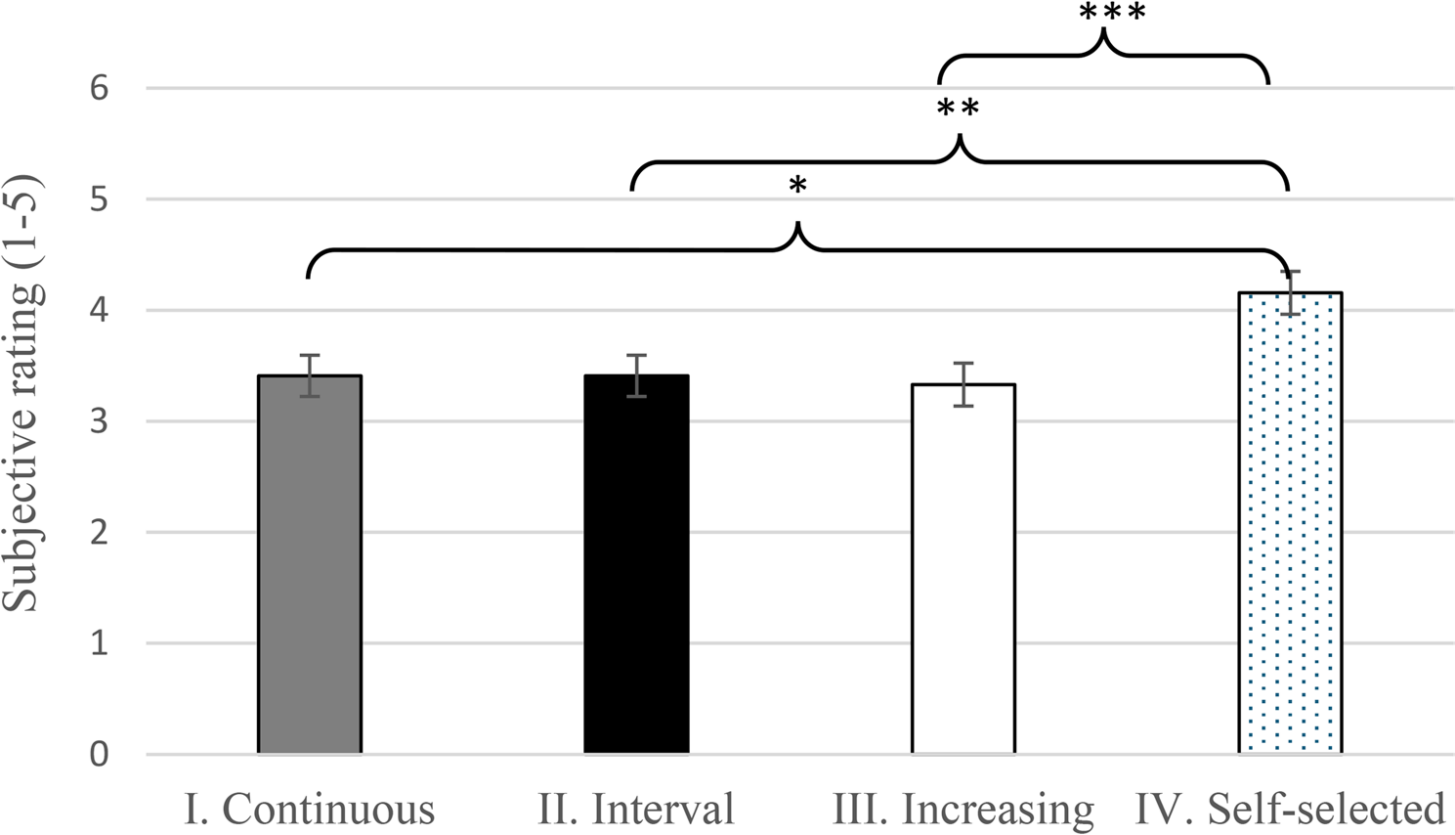
Adjusted subjective rating (on a scale of 1 to 5) of the warm-up types (mean, +/- SD) *** *p* = 0.001, ** *p* = 0.002, * *p* = 0.002. The complete model output underlying the estimated means and diagnostic plots can be found in Supplementary D

Participants rated the Self-selected WU the best with a mean subjective rating of 4.16 (95% CI 3.78–4.54) on a 1 to 5 scale. The other three WU types were rated significantly lower (Fig. 4).

Previous analysis showed that WU style was significantly associated with TT performance, as was lactate measured at the start. To investigate the potential effect of lactate at start on TT performance, we fit a mixed-effect linear model on TT performance with lactate as a fixed-effect predictor, and individual as the random-effect. Table 4 shows that higher lactate at start corresponds to a significantly lower TT performance. With a similar justification, the difference between peak and start lactate and this difference relative to peak lactate were also analysed. These showed the same fundamental association as lactate at start. Including HR_peak_ in the model did not change the results, and its effect was non-significant.

**Table 4:** Mixed-effects linear model for TT performance (W). Fixed-effects: lactate at start; random-effects: individual

Variable	Estimate	SE	d.f.	t-value	*p*-value
Constant	252.03	11.81			
Lactate at start (mmol/L)	-7.16	1.47	30.94	-4.884	< 0.001

As WU style was associated with both TT performance and lactate at the start of the TT, and TT performance was also associated with lactate at start, we fit a mixed-effects linear model on TT performance using both WU style and lactate at start as fixed-effect predictors, while keeping the individuals as a random-effect to account for baseline differences and correlation of measurements. As shown in Table 5, lactate at start has a negative significant effect on TT performance each mmol/L additional lactate is associated with a mean decrease of performance by about 7 watts, even accounting for differences in WU types. Conversely, controlling for lactate at start there are no significant differences between WU types in mean TT performance.

**Table 5:** Mixed-effects linear model for TT performance (W). Fixed-effects: WU style, lactate at start; random-effects: individual

Variable	Estimate	SE	d.f.	t-value	*p*-value
II. Interval – reference	253.08	12.18			
I. Continuous	-1.22	6.58	27.12	-0.185	0.855
III. Increasing	-0.95	8.36	27.47	-0.114	0.910
IV. Self-selected	-4.10	6.87	27.20	-0.597	0.555
Lactate at start (mmol/L)	-7.02	1.98	28.35	-3.553	**0.001**

Individual linear trajectories for the association of TT performance and lactate at start by WU style are shown in Supplementary Fig. 6.

**Fig. 5:**
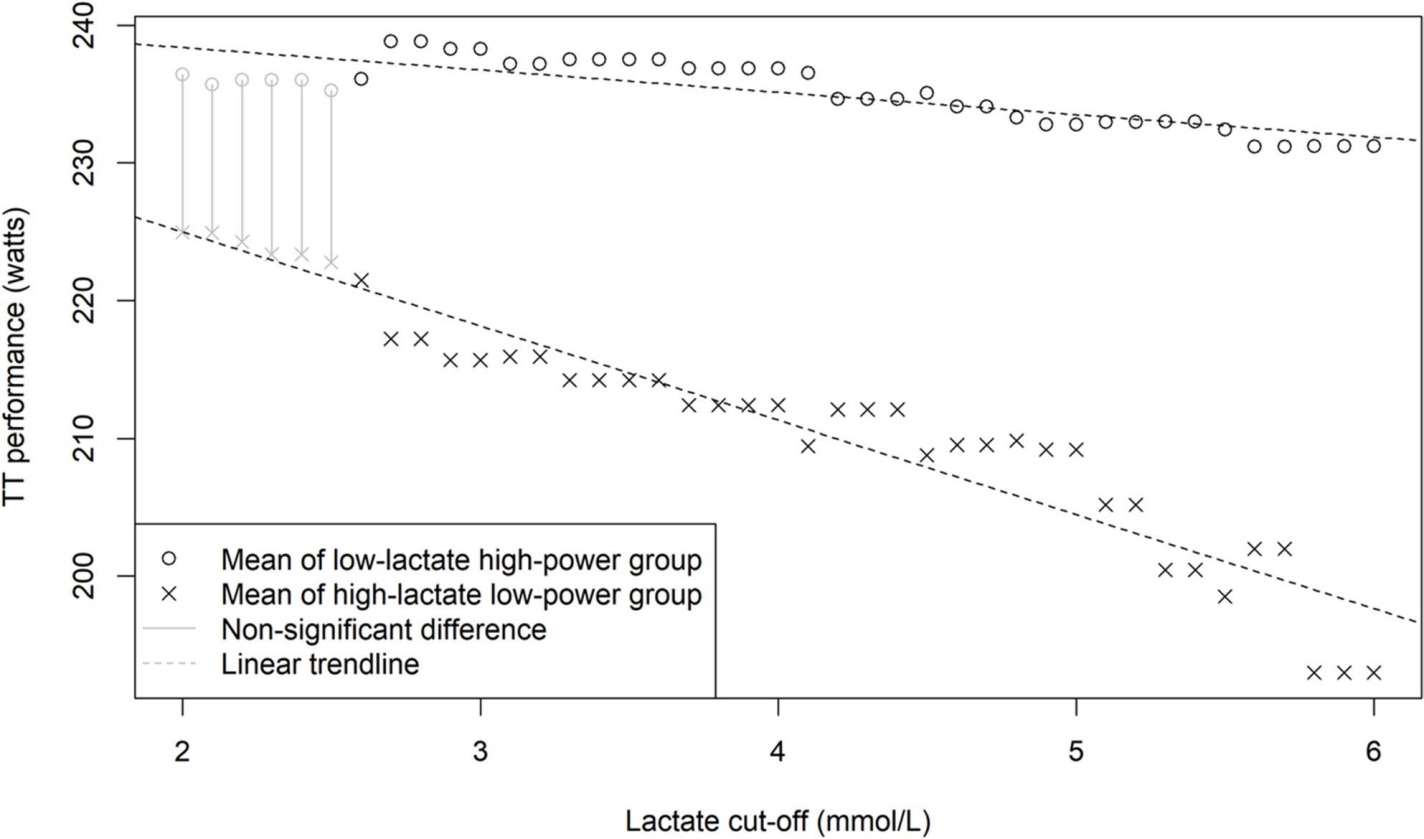
Adjusted mean TT performance of high- and low-lactate groups as a function of different lactate at start cut-off values

While a negative linear association between La_s_ and TT performance can be observed, a specific cut-off for lactate can also be identified, above which performance significantly declines. Figure 5 shows the comparisons of mean TT performances between high-lactate and low-lactate groups for multiple cut points in La_s_.

In general, a higher cut-off corresponds to a greater difference between the two groups, but this effect is only significant above a cut-off of 2.7 mmol/L lactate at start. The coefficient of the mixed-effects model shown in Table 6 shows the estimated difference between high- and low-lactate groups with a cut-off of 2.7 mmol/L lactate at start. The mean TT performance estimated by the model for those with a starting lactate under 2.7 mmol/L was 238.81 (95% CI 217.91–259.72) watts, while the mean for those with a starting lactate over 2.7 mmol/L was 217.21 (95% CI 196.22–238.20) watts. This effect is also observed and is significant regardless of WU style.

**Table 6:** Mixed-effects linear model for TT performance (watts). Fixed-effects: High/Low lactate at start; random-effects: individual

Variable	Estimate	SE	d.f.	t-value	*p*-value
Low-lactate group – reference	238.81	10.67	11.50		
High-lactate group	-21.60	5.82	31.61	-3.713	< 0.001

The complete model output can be found in the Supplementary G.

Table 7 summarizes the total and direct effects of lactate and WU style on TT performance. While Increasing WU is significantly worse than the Interval style without considering lactate, the difference becomes insignificant when controlling for lactate. Lactate’s effect size on TT performance and its significance remains unaltered when controlling for WU style. This indicates that lactate is mediating variable between WU style and TT performance.

**Table 7:** Total and direct fixed effects on TT performance (W). Random-effects: individual

Variable	Effect on TT performance (W)			
	Total effect (SE)	*p*-value	Direct effect (SE)	*p*-value
Lactate	-7.16 (1.47)	**< 0.001**	-7.02	**0.001**
II. Interval – reference				
I. Continuous	-7.91 (7.60)	0.304	-1.22 (6.58)	0.855
III. Increasing	-19.49 (7.82)	**0.017**	-0.95 (8.36)	0.910
IV. Self-selected	-11.97 (7.82)	0.135	-4.10 (6.87)	0.555

Direct effect of lactate from model with WU styles as control variable; direct effect of WU styles from model with lactate as control variable.

## Discussion

The present study investigated the effects of different WU protocols—Self-selected, Interval, Continuous, and Increasing WUs—on a 2-minute TT performance on kayak ergometer in elite Hungarian sprint kayakers.

Warm-up is known to induce various beneficial physiological effects, such as increased muscle and core temperature, improved neuromuscular activation, and favourable metabolic and cardiovascular changes, which together enhance performance [1, 3, 4]. However, the warm-up must be carefully dosed to avoid excessive fatigue [8]. Our results align with previous findings indicating that overly intense warm-ups, such as the Increasing WU protocol in our study, may elevate lactate and induce metabolic acidosis, which can impair performance [10]. The lack of significant differences between the Interval, Continuous, and Self-selected WUs supports the notion that individualized warm-up strategies may be equally effective, particularly in highly experienced athletes [12].

### Impact of WU types on 2-min TT performance

2-min TT performance reached the highest average power after the Interval-type WU. However, the Interval-type WU was significantly better only compared to the Increasing-type WU, while there was no significant difference in comparison to the Self-selected type WU, or to the Continuous-type WU.

This is in line with the study of Bishop et al. [11], where they found that canoers reached significantly higher average power output on supramaximal kayak ergometer performance after completing intermittent, high intensity rather than a continuous-type warm-up.

However, in our study significant difference was found only between the Interval and the Increasing WU. The Increasing WU was followed by the lowest average power at the 2-min TT performance while it generated the highest average lactate level at the start of the TT. The aim of the Increasing WU was to elevate both power output and HR, while the goal with the Interval WU was to reach high power output without increasing the HR above the HR of the IAT. Yet, the peak power achieved during the Increasing WU protocol matched the highest values reached during the Interval WU. In terms of HR, the goal of the Increasing WU was to reach 95% of the individual HR_max_. From a physiological perspective, reaching a high HR alongside high intensity likely resulted in spending more time in a high-stress zone, which may have contributed to increased fatigue [8]. Altogether, Increasing WU resulted in the lowest subsequent performance and was associated with the highest starting lactate concentration.

### Impact of warm-ups on starting lactate level

A key finding of this study was that the lactate concentration measured immediately before the TT significantly mediated the effects of WU type on performance. Lower pre-exercise lactate levels, observed after the Interval and Continuous WUs, correlated with higher TT performance, whereas the Increasing WU type resulted in the highest lactate concentration and correspondingly lower performance. This suggests that the Increasing WU overstressed the skeletal muscles, producing an early metabolic fatigue detrimental to sprint performance.

Interestingly, in the study by Bishop et al. [11], the interval-type warm-up resulted in higher lactate concentrations before the TT compared to the continuous warm-up. However, in their study, lactate level was < 2.0 mmol/L after the continuous warm-up, while lactate was > 3.0 mmol/L after the interval-type warm-up. It is necessary to highlight that in our study the highest lactate level (after the Increasing WU) was above 5 mmol/L while even the lowest lactate concentration (measured after the Interval WU) was above 2.0 mmol/L. Bishop et al. suggested that the metabolic acidaemia associated with elevated lactate following interval-type warm-ups might enhance oxygen delivery to the working muscles without negatively affecting oxygen uptake kinetics. However, in their previous study [10], it was already concluded that although a certain level of metabolic acidaemia may be advantageous by enhancing O_2_ kinetics, if the warm-up is too intense, the concomitant metabolic acidaemia may compromise performance by decreasing the anaerobic energy contribution.

This theory is consistent with recent publications underlining the importance of balancing activation and fatigue during warm-up to optimize PAPE [4, 9]. Given that phosphocreatine stores and acid-base imbalances can be relatively quickly restored during rest [8], warm-ups that elevate lactate excessively or fail to allow sufficient recovery may compromise the ergogenic benefits. Thus, managing lactate accumulation through the intensity and recovery structure of the warm-up is crucial to maximizing sprint kayak performance.

It is necessary to highlight that in our study there was only a 2-minute recovery between WUs and the start of the TT. This is rather on the short end since optimal recovery time is approximated around 5 min in the current literature [9]. However, in our study recovery time was chosen based on the consultation with the Hungarian Canoe Federation.

It is well-known that the recovery after warm-up highly affects the subsequent performance. In the study by Hajoglou et al. [26], it was observed that both easy and strenuous warm-up protocols, followed by a 2-minute rest, significantly enhanced 3-kilometer cycling time-trial performance compared to no warm-up. Christensen et al. [27] found that high-intensity warm-ups without appropriate recovery can reduce endurance performance in trained cyclists. These results indicate that a high lactate level prior to the start clearly has a negative effect on the subsequent maximal performance. Our findings further support that a 2-minutes pre-start interval is suboptimal and emphasizes the importance of an appropriately chosen recovery period following the warm-up. According to recent literature, a ~ 5-minute interval is recommended as the optimal recovery time, although the most advantageous elapsed time between warm-up and the start of the race might differ among different sport disciplines [9].

Our findings demonstrate that overly intense warm-ups, which excessively raise lactate levels, such as the Increasing WU, may impair subsequent kayak ergometer performance without sufficient recovery. This further supports the need to optimize warm-up intensity and duration to balance post-activation performance enhancement and fatigue management in sprint kayaking, similar to other endurance sports.

Evidence from our data indicates that lactate levels at the start not only significantly influence performance, but also eliminate differences between warm-up strategies once accounted for. This underscores the need for future studies employing formal mediation analysis to further examine lactate as a potential mediating factor.

Furthermore, we observed that increasing lactate levels were associated with reduced performance, with each 1 mmol/L rise corresponding to a 7 W decrease. A cut-off point of 2.7 mmol/L was also identified, above which performance declined significantly. In this context, the lack of significant differences among certain warm-up types may have been due to elevated lactate masking potential effects. Accordingly, future research should compare different warm-up strategies under conditions of optimized pre-race lactate levels.

### Impact of warm-up types on subjective rating

Interestingly, despite the Interval WU producing the highest average power, athletes rated the Self-selected WU with the highest subjective ranking and there was a significant difference between the Self-selected and the other three WU types. However, objective measures did not support this evaluation since TT performance was not the highest. This discrepancy between perceived effectiveness and physiological outcomes underscores the multifaceted nature of warm-up efficacy.

Athlete preference for self-selected warm-up may be influenced by psychological factors such as confidence, familiarity, and comfort, which have been shown to affect readiness and performance [3]. While some literature suggests that tailored warm-up protocols can optimize physiological responses [1], others support the autonomy of athletes to select their preferred routines without compromising performance [12].

Our findings suggest that allowing athlete input in warm-up design might have additional benefits, yet coaches should also consider objective physiological markers, such as lactate and HR, to guide warm-up programming effectively.

### Limitations

The sample size of participants was relatively small, which may limit the generalizability of the findings. Supplementary E shows the power curve for WU style differences on TT performance as a function of the number athletes. Because of the lack of statistical significance, these findings should be interpreted cautiously rather than as evidence of equivalence. Additionally, the Self-selected WU was not closely monitored or controlled, and a more detailed analysis of this warm-up type is planned for a future study. Furthermore, the assessment of subjective ratings relied exclusively on the Likert scale, which is not sufficiently detailed. The recovery time between the WUs and the TT was only 2 min, whereas the current literature recommends a longer interval of approximately 5 min. Another limitation is that the tests were performed on a kayak ergometer rather than on water, which may have influenced the results. Some athletes might not have been able to exert maximal effort in a non-competitive, laboratory setting, which could affect the ecological validity of the findings.

## Conclusions

Among the four different types of warm-ups, the Interval WU was the only one to show a significant improvement in 2-min TT performance, but this was only significant when compared to the Increasing WU. However, the performance after Self-selected WU was not significantly different from the Interval WU, and participants rated the Self-selected WU significantly higher than any other WU types.

A clear connection was found between starting lactate levels and TT performance: warm-ups that resulted in lower lactate levels before the TT were associated with higher TT performance. Although starting lactate levels were never below 2.0 mmol/L for any WU type, the highest starting lactate was linked to the lowest performance.

Since Self-selected WU type received the highest subjective rating, had a significantly lower starting lactate level compared to the overly stressing Increasing WU style, and there were no significant differences in performance between the Interval and Self-selected WUs, it appears that allowing elite athletes to choose their own WU may be acceptable. Accordingly, athletes may be allowed to retain their habitual, self-selected warm-up, provided that certain objective criteria are met. This preference comes with the following recommendation:

- Lactate concentration at the start of the race is suggested to remain below 2.7 mmol/L.
- It is advised that warm-up approach race pace or maximal power output at least three times, maintaining it for no longer than 8-10 seconds each time.
- Monitoring heart rate to remain below the individual anaerobic threshold (HR ≤ IAT) throughout the warm-up, even when reaching high intensities, may help prevent excessive lactate accumulation.
- Adhering to the recovery interval (~5min) recommended in the current literature [9] is crucial for an efficient, yet not overstressing warm-up.

## Supplementary Information

Supplementary Material 1.

## Data Availability

The data belongs to the Katalin Kovács National Canoe Sports Academy and are not openly available because of sensitivity reasons but are available from the corresponding author upon reasonable request.

## Appendix

Dry-land warm-up developed specifically for kayakers https://www.youtube.com/watch?v=xNAtVyVp3Tg

## Abbreviations

AT: Anaerobic Threshold
GXT: Graded Exercise Test
HR: Heart Rate
HR_peak_: Peak Heart Rate
IAT: Individual Anaerobic Threshold
TT: Time Trial
La_peak_: Peak Lactate Concentration
La_s_: Lactate Concentration at Start of TT
LT: Lactate Threshold
PAPE: Post–Activation Performance Enhancement
P_max_: Maximal Power
WU: Warm–up
