# Supplementary material for: The effect of self-selected versus standardised warm-ups on kayak ergometer performance in Hungarian elite kayakers: a randomized controlled crossover trial
Source: BMC Sports Sci Med Rehabil. 2026 Feb 5;18:112. doi: 10.1186/s13102-026-01555-6 (PMC12964868; doi:10.1186/s13102-026-01555-6)
Supplement: Supplementary file 1 — Supplementary Material 1. [file 13102_2026_1555_MOESM1_ESM.docx]

Supplementary material

Supplementery A. Model output, diagnostic plots, and effect size estimates for the model on TT performance

Supplementery Table 1 Complete model output and diagnostic plots for the mixed-effects linear model on TT performance.

|  | **TT performance** (W) | | | | |
| --- | --- | --- | --- | --- | --- |
| *Predictors* | *Estimates* | *CI* | *Statistic* | *p* | *df* |
| II. Interval WU - reference | 237.82 | 214.98 – 260.66 | 21.12 | **<0.001** | 36.00 |
| I. Continuous WU | -7.91 | -23.28 – 7.46 | -1.04 | 0.304 | 36.00 |
| III. Increasing WU | -19.49 | -35.36 – -3.63 | -2.49 | **0.017** | 36.00 |
| IV. Self-selected WU | -11.97 | -27.84 – 3.89 | -1.53 | 0.135 | 36.00 |
| **Random Effects** | | | | | |
| σ^2^ | 315.92 | | | | |
| τ_00_ _Athlete_ | 1079.12 | | | | |
| ICC | 0.77 | | | | |
| N _Athlete_ | 11 | | | | |
| Observations | 42 | | | | |
| Marginal R^2^ / Conditional R^2^ | 0.035 / 0.781 | | | | |
| AIC | 374.271 | | | | |


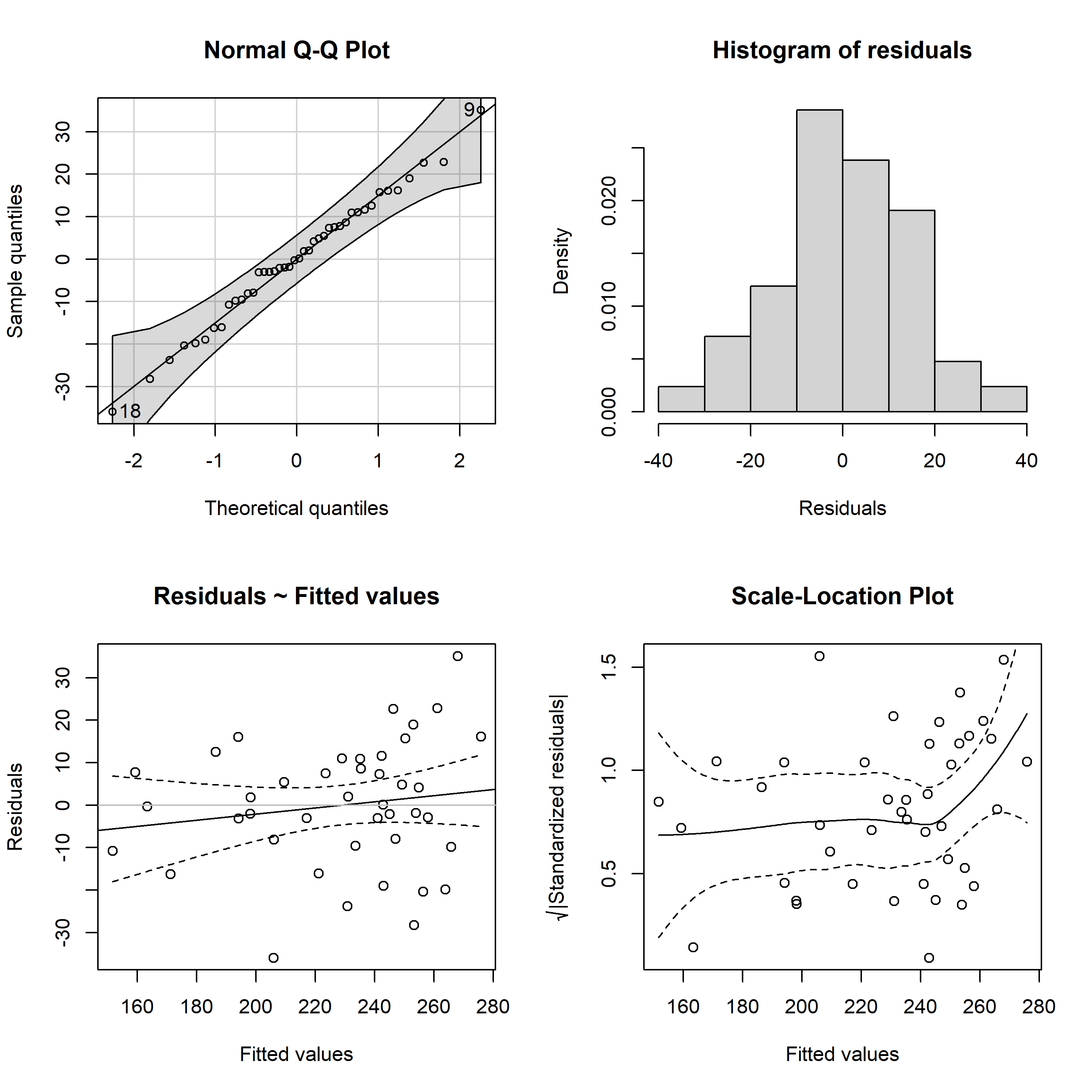


Supplementery Fig. 1 Diagnostic plots for the mixed-effects linear model on TT performance.

Supplementery Table 2 Cohen’s d effect sizes for the mixed-effects linear model on TT performance.

| Comparisons | Effect size | SE | d.f. | 95% CI |
| --- | --- | --- | --- | --- |
| WU 2 effect - WU 1 effect | 0.445 | 0.430 | 28.023 | (-0.435 - 1.325) |
| WU 2 effect - WU 3 effect | 1.097 | 0.440 | 28.023 | (0.195 - 1.999) |
| WU 2 effect - WU 4 effect | 0.674 | 0.443 | 28.023 | (-0.234 - 1.582) |
| WU 1 effect - WU 3 effect | 0.652 | 0.443 | 28.023 | (-0.256 - 1.560) |
| WU 1 effect - WU 4 effect | 0.229 | 0.440 | 28.023 | (-0.673 - 1.131) |
| WU 3 effect - WU 4 effect | -0.423 | 0.457 | 28.136 | (-1.359 - 0.513) |

WU 1: I. Continuous, WU 2: II. Interval, WU 3: III. Increasing, WU 4: IV. Self-selected.

Supplementary B. Model output, diagnostic plots, and effect size estimates for the model on peak heart rate

Supplementary Table 3 Complete model output and diagnostic plots for the mixed-effects linear model on peak heart rate.

|  | **Peak heart rate** (beat/min) | | | | |
| --- | --- | --- | --- | --- | --- |
| *Predictors* | *Estimates* | *CI* | *Statistic* | *p* | *df* |
| II. Interval WU - reference | 191.45 | 185.97 – 196.94 | 70.73 | **<0.001** | 36.00 |
| I. Continuous WU | 2.00 | -2.51 – 6.51 | 0.90 | 0.374 | 36.00 |
| III. Increasing WU | 3.78 | -0.87 – 8.42 | 1.65 | 0.108 | 36.00 |
| IV. Self-selected WU | -2.93 | -7.58 – 1.72 | -1.28 | 0.210 | 36.00 |
| **Random Effects** | | | | | |
| σ^2^ | 27.16 | | | | |
| τ_00_ _Athlete_ | 53.43 | | | | |
| ICC | 0.66 | | | | |
| N _Athlete_ | 11 | | | | |
| Observations | 42 | | | | |
| Marginal R^2^ / Conditional R^2^ | 0.070 / 0.687 | | | | |
| AIC | 276.029 | | | | |


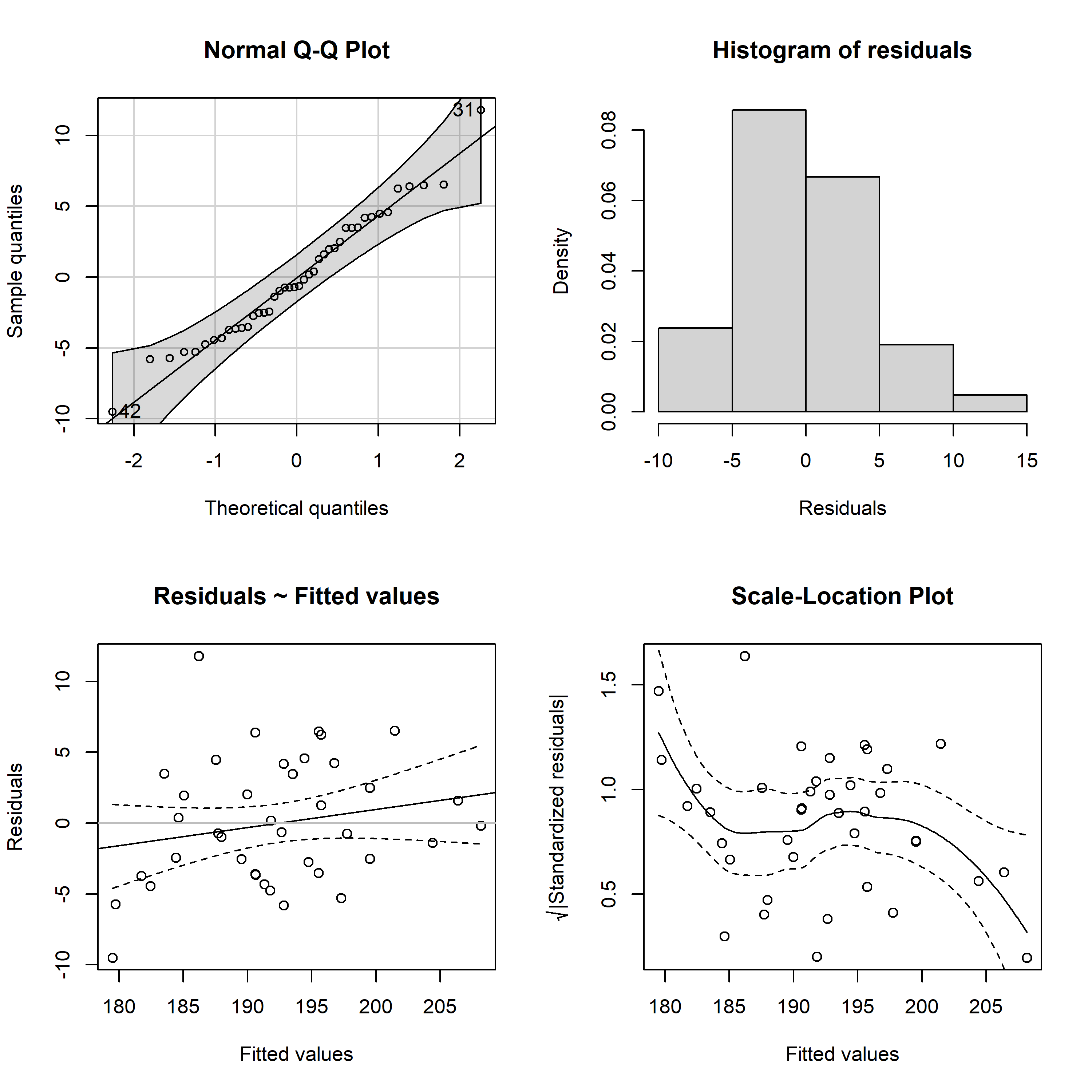


Supplementary Fig. 2 Diagnostic plots for the mixed-effects linear model on peak heart rate.

Supplementary Table 4 Cohen’s d effect sizes for the mixed-effects linear model on peak heart rate.

| Comparisons | Effect size | SE | d.f. | 95% CI |
| --- | --- | --- | --- | --- |
| WU 2 effect - WU 1 effect | -0.384 | 0.427 | 28.039 | (-1.258 - 0.490) |
| WU 2 effect - WU 3 effect | -0.724 | 0.443 | 28.039 | (-1.632 - 0.184) |
| WU 2 effect - WU 4 effect | 0.562 | 0.445 | 28.039 | (-0.350 - 1.473) |
| WU 1 effect - WU 3 effect | -0.341 | 0.442 | 28.039 | (-1.246 - 0.565) |
| WU 1 effect - WU 4 effect | 0.945 | 0.443 | 28.039 | (0.037 - 1.854) |
| WU 3 effect - WU 4 effect | 1.286 | 0.454 | 28.220 | (0.355 - 2.217) |

WU 1: I. Continuous, WU 2: II. Interval, WU 3: III. Increasing, WU 4: IV. Self-selected.

Supplementary C. Model output, diagnostic plots, and effect size estimates for the model on lactate at start

Supplementary Table 5 Complete model output and diagnostic plots for the mixed-effects linear model on lactate at start.

|  | **Lactate at start** (mmol/L) | | | | |
| --- | --- | --- | --- | --- | --- |
| *Predictors* | *Estimates* | *CI* | *Statistic* | *p* | *df* |
| II. Interval WU - reference | 2.17 | 1.26 – 3.09 | 4.81 | **<0.001** | 36.00 |
| I. Continuous WU | 0.95 | -0.25 – 2.16 | 1.60 | 0.117 | 36.00 |
| III. Increasing WU | 2.72 | 1.48 – 3.96 | 4.46 | **<0.001** | 36.00 |
| IV. Self-selected WU | 1.11 | -0.13 – 2.34 | 1.81 | 0.078 | 36.00 |
| **Random Effects** | | | | | |
| σ^2^ | 1.94 | | | | |
| τ_00_ _Athlete_ | 0.31 | | | | |
| ICC | 0.14 | | | | |
| N _Athlete_ | 11 | | | | |
| Observations | 42 | | | | |
| Marginal R^2^ / Conditional R^2^ | 0.301 / 0.397 | | | | |
| AIC | 159.195 | | | | |


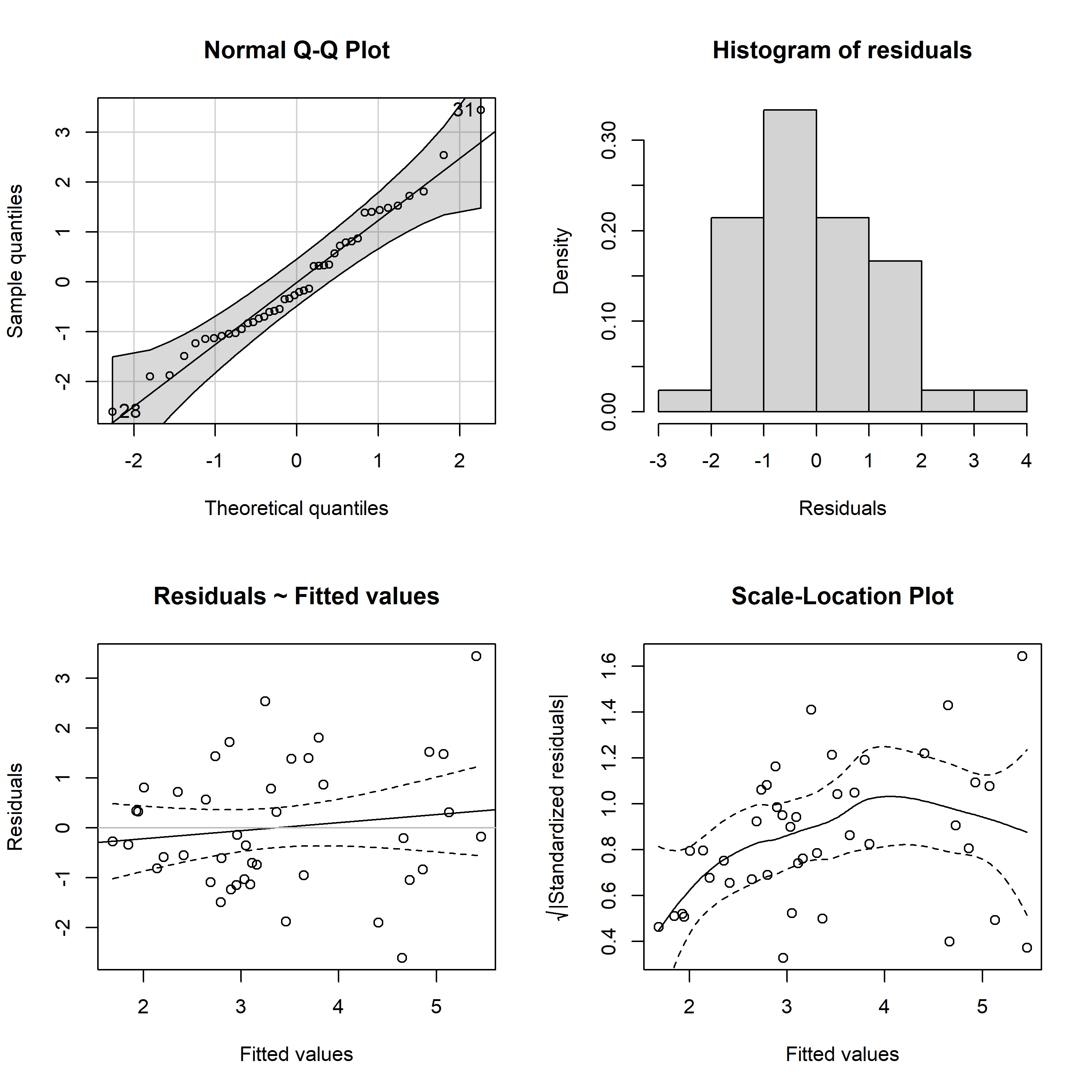


Supplementary Fig. 3 Diagnostic plots for the mixed-effects linear model on lactate at start.

Supplementary Table 6 Cohen’s d effect sizes for the mixed-effects linear model on lactate at start.

| Comparisons | Effect size | SE | d.f. | 95% CI |
| --- | --- | --- | --- | --- |
| WU 2 effect - WU 1 effect | -0.684 | 0.434 | 28.206 | (-1.573 - 0.204) |
| WU 2 effect - WU 3 effect | -1.952 | 0.440 | 28.206 | (-2.853 - -1.052) |
| WU 2 effect - WU 4 effect | -0.794 | 0.449 | 28.206 | (-1.713 - 0.125) |
| WU 1 effect - WU 3 effect | -1.268 | 0.452 | 28.206 | (-2.194 - -0.342) |
| WU 1 effect - WU 4 effect | -0.110 | 0.439 | 28.206 | (-1.009 - 0.790) |
| WU 3 effect - WU 4 effect | 1.158 | 0.468 | 28.938 | (0.202 - 2.115) |

WU 1: I. Continuous, WU 2: II. Interval, WU 3: III. Increasing, WU 4: IV. Self-selected.

Supplementary D. Model output, diagnostic plots, and effect size estimates for the model on subjective rating of warm-up

Supplementary Table 7 Complete model output and diagnostic plots for the mixed-effects linear model on subjective rating of warm-up.

|  | **Subjective rating** (1-5 Likert scale) | | | | |
| --- | --- | --- | --- | --- | --- |
| *Predictors* | *Estimates* | *CI* | *Statistic* | *p* | *df* |
| II. Interval WU - reference | 3.41 | 3.03 – 3.79 | 18.36 | **<0.001** | 36.00 |
| I. Continuous WU | -0.00 | -0.44 – 0.44 | -0.00 | 1.000 | 36.00 |
| III. Increasing WU | -0.08 | -0.53 – 0.37 | -0.36 | 0.724 | 36.00 |
| IV. Self-selected WU | 0.75 | 0.29 – 1.20 | 3.34 | **0.002** | 36.00 |
| **Random Effects** | | | | | |
| σ^2^ | 0.26 | | | | |
| τ_00_ _Athlete_ | 0.12 | | | | |
| ICC | 0.31 | | | | |
| N _Athlete_ | 11 | | | | |
| Observations | 42 | | | | |
| Marginal R^2^ / Conditional R^2^ | 0.228 / 0.469 | | | | |
| AIC | 88.152 | | | | |


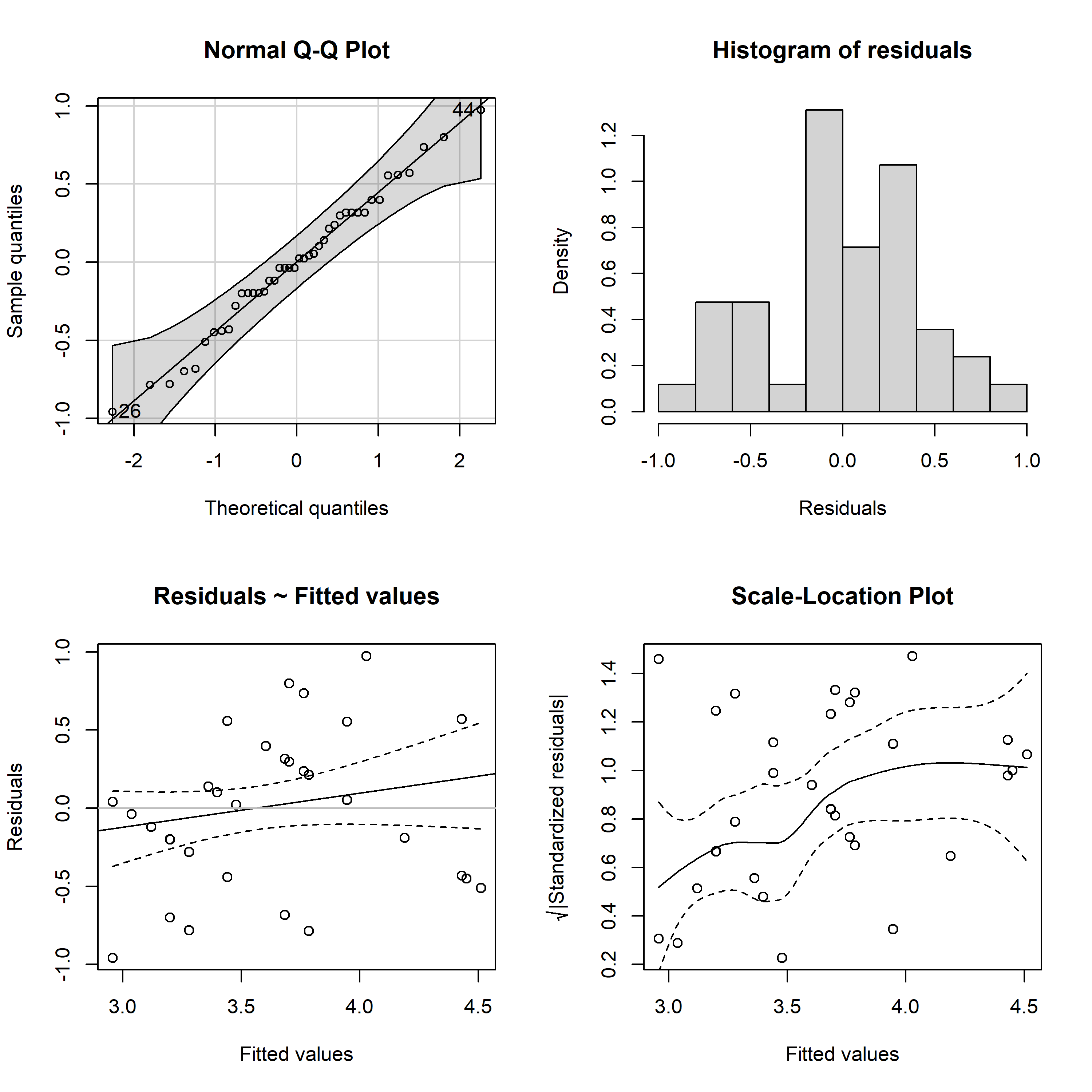


Supplementary Fig. 4 Diagnostic plots for the mixed-effects linear model on subjective rating of warm-up.

Supplementary Table 8 Cohen’s d effect sizes for the mixed-effects linear model on subjective rating of warm-up.

| Comparisons | Effect size | SE | d.f. | 95% CI |
| --- | --- | --- | --- | --- |
| WU 2 effect - WU 1 effect | 0.000 | 0.426 | 28.121 | (-0.873 - 0.873) |
| WU 2 effect - WU 3 effect | 0.156 | 0.440 | 28.121 | (-0.745 - 1.057) |
| WU 2 effect - WU 4 effect | -1.465 | 0.450 | 28.121 | (-2.386 - -0.543) |
| WU 1 effect - WU 3 effect | 0.156 | 0.440 | 28.121 | (-0.745 - 1.057) |
| WU 1 effect - WU 4 effect | -1.465 | 0.450 | 28.121 | (-2.386 - -0.543) |
| WU 3 effect - WU 4 effect | -1.621 | 0.460 | 28.613 | (-2.561 - -0.680) |

WU 1: I. Continuous, WU 2: II. Interval, WU 3: III. Increasing, WU 4: IV. Self-selected.

Supplementary E. Power and sample size calculation for TT performance

Supplementary Table 9 Power to detect the observed TT performance difference between WU styles for 11 and 14 athletes.

| Number of subjects | Power | | | | | |
| --- | --- | --- | --- | --- | --- | --- |
|  | WU 1 vs. WU 2 | WU 1 vs. WU 3 | WU 1 vs. WU 4 | WU 2 vs. WU 3 | WU 2 vs. WU 4 | WU 3 vs. WU 4 |
| 11 | 0.167 | 0.332 | 0.081 | 0.718 | 0.334 | 0.168 |
| 14 | 0.204 | 0.391 | 0.096 | 0.836 | 0.420 | 0.203 |


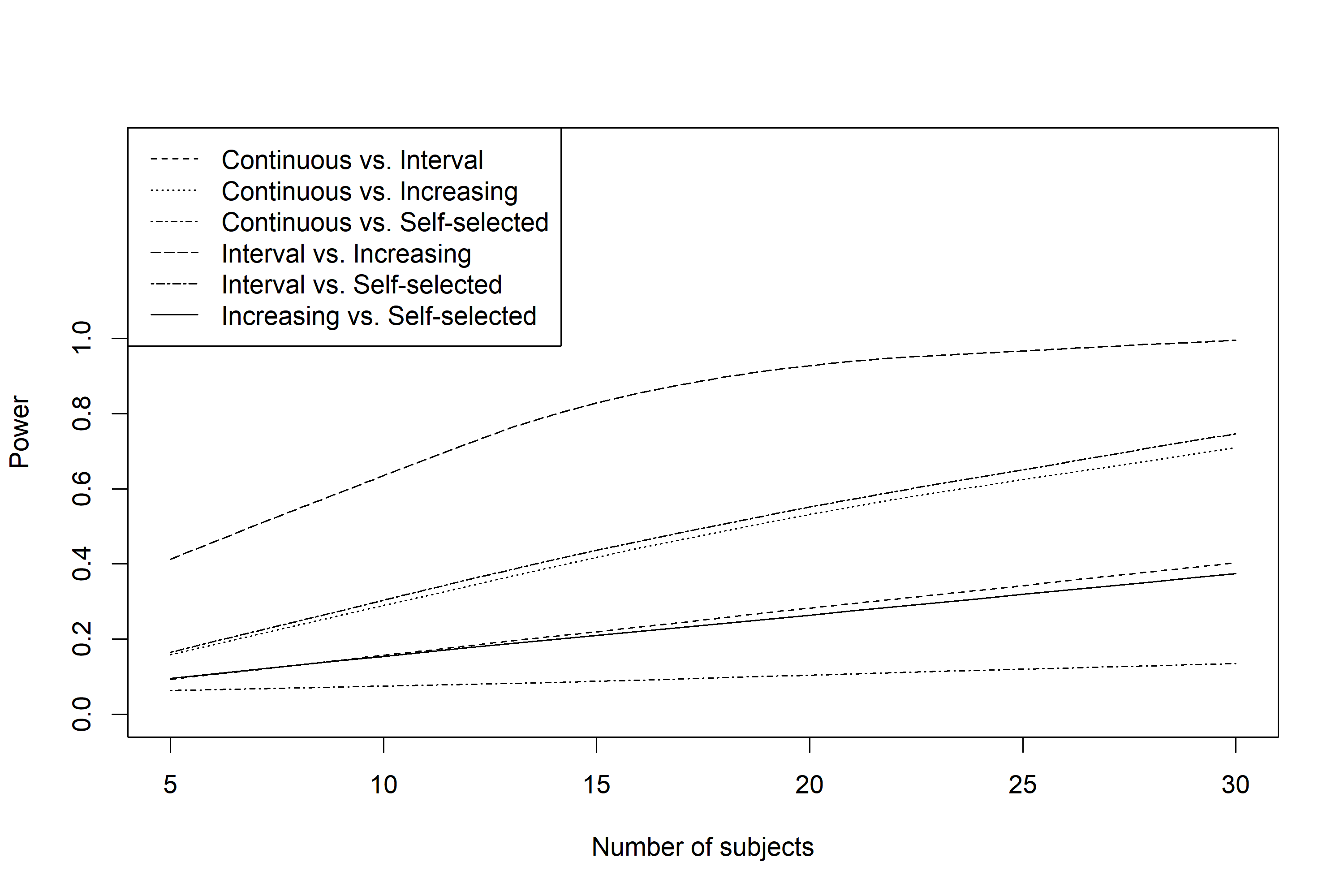


Supplementary Fig. 5 Power as a function of the number of athletes to detect the observed TT performance difference between WU styles.

Supplementary F. Individual trajectories for the association of TT performance and lactate across WU styles.


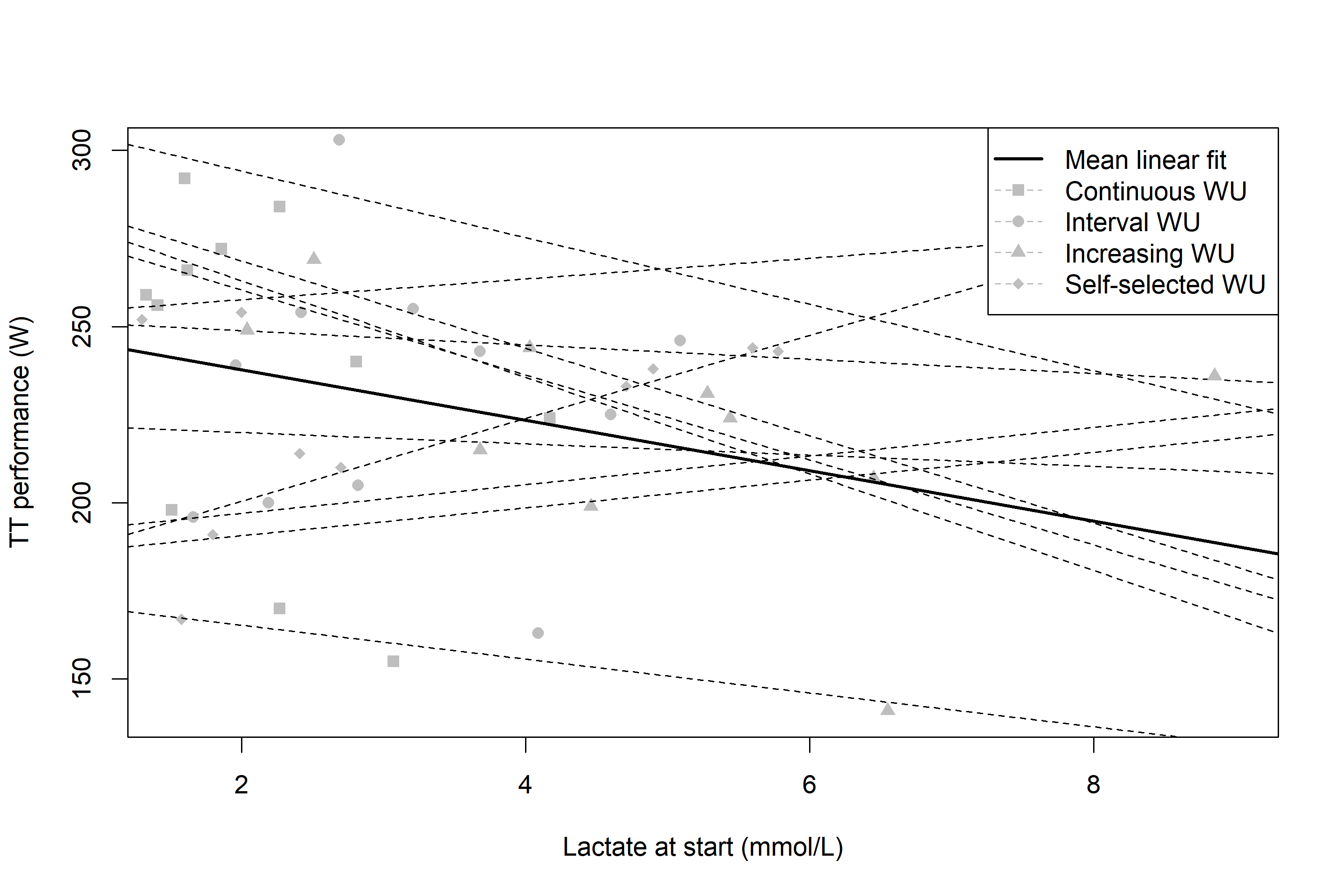


Supplementary Fig. 6 Individual differences in TT performance as a function of lactate at start across WU styles.

Supplementary G. Association of lactate at start cut-off and TT performance.

Supplementary Table 10 Complete model output and diagnostic plots for the mixed-effects linear model on TT performance for the high-lactate and low-lactate groups

|  | **TT performance** (watts) | | | | |
| --- | --- | --- | --- | --- | --- |
| *Predictors* | *Estimates* | *CI* | *Statistic* | *p* | *df* |
| Low-lactate group - reference | 238.81 | 217.22 – 260.40 | 22.39 | **<0.001** | 38.00 |
| High-lactate group | -21.60 | -33.38 – -9.82 | -3.71 | **0.001** | 38.00 |
| **Random Effects** | | | | | |
| σ^2^ | 249.18 | | | | |
| τ_00_ _Athlete_ | 1097.17 | | | | |
| ICC | 0.81 | | | | |
| N _Athlete_ | 11 | | | | |
| Observations | 42 | | | | |
| Marginal R^2^ / Conditional R^2^ | 0.082 / 0.830 | | | | |
| AIC | 376.717 | | | | |
